# An Alphaherpesvirus Exploits Antimicrobial β-Defensins To Initiate Respiratory Tract Infection

**DOI:** 10.1128/JVI.01676-19

**Published:** 2020-03-31

**Authors:** Jolien Van Cleemput, Katrien C. K. Poelaert, Kathlyn Laval, Nathalie Vanderheijden, Maarten Dhaenens, Simon Daled, Filip Boyen, Frank Pasmans, Hans J. Nauwynck

**Affiliations:** a Department of Virology, Parasitology and Immunology, Faculty of Veterinary Medicine, Ghent University, Merelbeke, Belgium; b Department of Molecular Biology, Princeton University, Princeton, New Jersey, USA; c Fox Chase Cancer Center, Philadelphia, Pennsylvania, USA; d Department of Pharmaceutics, Faculty of Pharmaceutical Sciences, Ghent University, Ghent, Belgium; e Department of Pathology, Bacteriology and Poultry Diseases, Faculty of Veterinary Medicine, Ghent University, Merelbeke, Belgium; Northwestern University

**Keywords:** β-defensins, alphaherpesvirus, equine herpesvirus, immune evasion, respiratory tract

## Abstract

β-Defensins protect the respiratory tract against the myriad of microbial pathogens entering the airways with each breath. However, this potentially hostile environment is known to serve as a portal of entry for herpesviruses. The lack of suitable respiratory model systems has precluded understanding of how herpesvirus virions overcome the abundant mucosal β-defensins during host invasion. We demonstrate how a central alphaherpesvirus, equine herpesvirus type 1 (EHV1), actually exploits β-defensins to invade its host and initiate viral spread. The equine β-defensins (eBDs) eBD1, -2, and -3 were produced and secreted along the upper respiratory tract. Despite the marked antimicrobial action of eBD2 and -3 against many bacterial and viral pathogens, EHV1 virions were resistant to eBDs through the action of the viral glycoprotein M envelope protein. Pretreatment of EHV1 virions with eBD2 and -3 increased the subsequent infection of rabbit kidney (RK13) cells, which was dependent on viral N-linked glycans. eBD2 and -3 also caused the aggregation of EHV1 virions on the cell surface of RK13 cells. Pretreatment of primary equine respiratory epithelial cells (EREC) with eBD1, -2, and -3 resulted in increased EHV1 virion binding to and infection of these cells. EHV1-infected EREC, in turn, showed an increased production of eBD2 and -3 compared to that seen in mock- and influenza virus-infected EREC. In addition, these eBDs attracted leukocytes, which are essential for EHV1 dissemination and which serve as latent infection reservoirs. These novel mechanisms provide new insights into herpesvirus respiratory tract infection and pathogenesis.

**IMPORTANCE** How herpesviruses circumvent mucosal defenses to promote infection of new hosts through the respiratory tract remains unknown due to a lack of host-specific model systems. We used the alphaherpesvirus equine herpesvirus type 1 (EHV1) and equine respiratory tissues to decipher this key event in general alphaherpesvirus pathogenesis. In contrast to several respiratory viruses and bacteria, EHV1 resisted potent antimicrobial equine β-defensins (eBDs) eBD2 and eBD3 by the action of glycoprotein M. Instead, eBD2 and -3 facilitated EHV1 particle aggregation and infection of rabbit kidney (RK13) cells. In addition, virion binding to and subsequent infection of respiratory epithelial cells were increased upon preincubation of these cells with eBD1, -2, and -3. Infected cells synthesized eBD2 and -3, promoting further host cell invasion by EHV1. Finally, eBD1, -2, and -3 recruited leukocytes, which are well-known EHV1 dissemination and latency vessels. The exploitation of host innate defenses by herpesviruses during the early phase of host colonization indicates that highly specialized strategies have developed during host-pathogen coevolution.

## Abstract (precis)

How herpesviruses circumvent mucosal defenses to promote infection of new hosts through the respiratory tract remains unknown due to a lack of host-specific model systems. We used the alphaherpesvirus equine herpesvirus type 1 (EHV1) and equine respiratory tissues to decipher this key event in general alphaherpesvirus pathogenesis. In contrast to several respiratory viruses and bacteria, EHV1 resisted potent antimicrobial equine β-defensins (eBDs) eBD2 and eBD3 by the action of glycoprotein M. Instead, eBD2 and -3 facilitated EHV1 particle aggregation and infection of rabbit kidney (RK13) cells. In addition, virion binding to and subsequent infection of respiratory epithelial cells were increased upon preincubation of these cells with eBD1, -2, and -3. Infected cells synthesized eBD2 and -3, promoting further host cell invasion by EHV1. Finally, eBD1, -2, and -3 recruited leukocytes, which are well-known EHV1 dissemination and latency vessels. The exploitation of host innate defenses by herpesviruses during the early phase of host colonization indicates that highly specialized strategies have developed during host-pathogen coevolution.

## INTRODUCTION

The Red Queen hypothesis postulates an ever-escalating evolutionary arms race between a virus and its host. Understanding the resulting virulence mechanisms is key in developing efficient disease mitigation. Herpesviruses have evolved to infect most vertebrates with noteworthy host specificity (1). Herpesvirus genomes are relatively large compared to many other viral genomes and encode a variety of novel gene products, including many virion envelope membrane proteins. This arsenal of membrane proteins has many functions, including virion attachment and entry, as well as engagement and modulation of host defense systems. These proteins enable the efficient colonization of new hosts, enabling the characteristic capacity of herpesvirus genomes to persist in the host population via the establishment of a lifelong infection with periods of latency and viral reactivation (2).

Although the vertebrate respiratory mucosa is often a portal of entry for herpesvirus virions, despite nearly a century of herpesvirus research, the mechanisms involved in the early interaction between virus particles and the host respiratory tract remain poorly understood (3–5). The respiratory mucosa is a hostile environment for microbes. Indeed, the respiratory epithelium harbors important antimicrobial and immunomodulatory peptides, including β-defensins (BDs). BDs are small cationic peptides that are characterized by three different intramolecular disulfide bonds pairing three antiparallel β strands (6–10). BDs act as efficient direct microbial killing agents by permeabilizing biological membranes and shielding pathogen ligands (7, 11–15). In addition, BDs aid in mounting the adaptive immune response by attracting immune cells to the site of infection (16, 17). How herpesvirus particles deal with these BDs during host colonization remains largely obscure. Understanding how herpesvirus infection bypasses or modulates the hostile respiratory environment could reveal new approaches for viral infection and transmission control. Indeed, prevention of infection is a key target to break the herpetic infection cycle, as latent infections are nearly impossible to clear (18, 19).

A major limitation in herpesvirus pathogenesis research is the lack of appropriate models. Most studies are conducted in continuous cell lines or in mice. Experimental infections in these systems do not adequately represent the *in vivo* situation. Here, we used the horse (Equus caballus) and the horse-specific alphaherpesvirus, equine herpesvirus type 1 (EHV1), as our study system. The EHV1 genome is closely related to other alphaherpesvirus genomes, and EHV1 likely shares many common attributes of infection and pathogenesis with other alphaherpesviruses (1). Here, we demonstrate how EHV1 establishes infection of the respiratory mucosa in the presence of antimicrobial BDs. We demonstrate how EHV1 circumvents the antimicrobial action of BDs and, instead, exploits their presence to facilitate mucosal infection and viral spread.

## RESULTS

### Characterization of synthetic eBDs.

The equine BDs (eBDs) were named here according to their homologs in humans, which were analyzed by BLAST analysis and which can be found in the NCBI database (Table 1). The sequences of the human BD1 (hBD1), hBD2, and hBD3 immunogens used for antibody production were >75% homologous to the respective sequences present in eBD1, eBD2, and eBD3 (Abcam, proprietary information).

**TABLE 1:** Information on equine β-defensins

Name	Amino acid sequence^*a*^	UniProt accession no.	NCBI gene name (accession no.)	Alternative peptide names (references)
eBD1	MRAPYFLLLTLCLFCCQMSSGVGYLTGLGHRSDHYICARSGGTCHFSSCPLFTKIEGTCYGGKAKCCL	N.D.^*b*^	DEFB1 (XM_005606422)	N.D.
eBD2	MRILHFLLAFLIVFLLPVPGFTAGIETSFSCSQNGGFCISPKCLPGSKQIGTCILPGSKCCRKK	Q865P6	DEFB4B (NM_001081887)	Defb1 (55, 63–69), Defb4B
eBD3	MRIHFLLFALLFLFLMPVPGNGGIINMLQKSYCKIRKGRCALLGCLPKEEQIGSCSVSGRKCCRKKK	Q0W9P9	BD103A (XM_003364245)	BD103A (69, 70)

a The signal peptide (not underlined) is followed by the synthesized peptide sequence (underlined).

b N.D., not determined.

**FIG 1:**
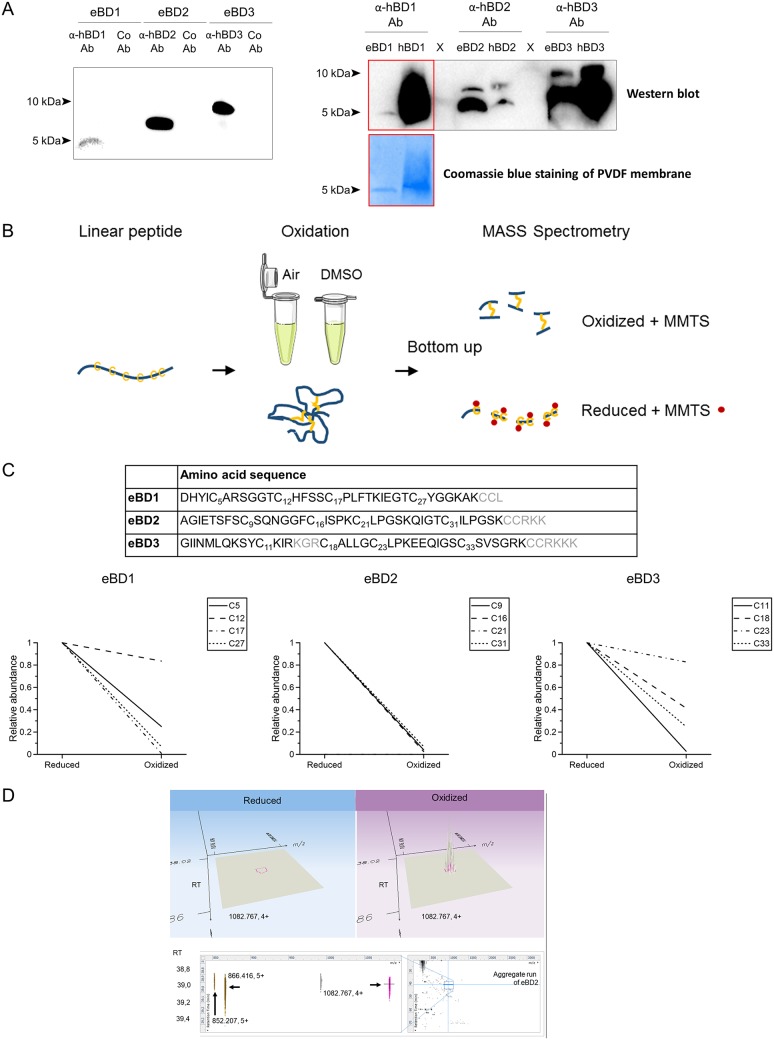
Validation of synthetic peptides and BD antibodies. (A) (Left) Western blot analysis of synthetic eBD1, -2, and -3 using the anti-human BD antibodies (α-hBD Ab) or isotype control antibodies (Co Ab). (Right) Western blotting to determine the cross-reactivity of the sera between eBDs and hBDs (top) and Coomassie blue staining of a Western blot membrane of eBD1 and hBD1 (bottom). The sizes (in kilodaltons) of the molecular weight markers are shown. (B) Work flow used to validate eBD oxidation via LC-MS analysis. Commercially synthesized eBD1, -2, and -3 (shown in blue) were folded through air oxidation (eBD2 and -3) or DMSO oxidation (eBD1). Disulfide bonds between cysteine molecules are shown in yellow. Next, bottom-up analysis was performed in triplicate to confirm oxidation of the eBDs and quantify the relative abundance of disulfide bridges in the oxidized versus reduced forms. Free cysteine molecules (yellow) in the reduced and oxidizede eBD1, -2, and -3 mixtures were alkylated by adding methyl methanethiosulfonate (MMTS; red dots) prior to overnight digestion in trypsin. The peptides were then identified through LC-ESI-MS. (C) (Top) Amino acid sequences of eBD1, -2, and -3. Amino acids indicated in gray were not covered in the MS bottom-up analysis. (Bottom) Relative abundance of disulfide bridge formation in the reduced and oxidized eBD1, -2, and -3 mixture. (D) Detection of the undigested oxidized form of eBD2. The small region of interest of the aggregate run from the eBD2 analysis is shown in three dimensions, comprising *m/z*, retention time (RT), and intensity as the dimensions. (Top) The signal of 1,082.767 (4+) is delineated in pink at a retention time of 39.0 min in both the reduced and the oxidized forms. (Bottom) Two-dimensional representations (retention time versus *m/z*) of extended regions of the LC-MS are shown. (Left) The 4+ form is highlighted in pink coeluting with its 5+ form (866.416), and the 5+ minus alanine (852.207) form is highlighted in brown. (Right) Complete overview of the aggregate LC-MS run (i.e., joint representation of the six runs on eBD2), with the region of interest depicted by the zoom box.

SDS-PAGE and Coomassie blue staining of the peptides revealed a stepwise pattern in eBD separation, where eBD1 migrates the fastest, followed by eBD2 and eBD3. The antibodies against hBD1 to hBD3, but not the isotype control antibody, immunoreacted with the respective eBDs, as shown in Fig. 1A, left. The cross-reactivity of the antibodies between eBD1 to eBD3 and hBD1 to hBD3 was assessed using Western blotting, which indicated that eBD2 and -3 could be detected to a similar extent as their human counterparts (Fig. 1A, right). Staining of the Western blot of eBD1 was rather difficult, as the low-molecular-weight protein detached quickly from the polyvinylidene difluoride (PVDF) membrane, even after glutaraldehyde fixation (20). Indeed, Coomassie blue staining of the Western blot membrane showed a much thinner protein band in the eBD1 lane than in the hBD1 lane. The same comment applies to the difference observed between eBD2 and hBD2. Still, immunofluorescence staining of human respiratory tissues derived from a previous study (5) showed similar distribution patterns and intensities, indicating that the antibodies detect human and equine BDs in a similar manner (data not shown).

The work flow used for liquid chromatography-mass spectrometry (LC-MS) analysis is shown in Fig. 1B. The *in vitro*-oxidized peptides were either reduced by dithiothreitol (DTT) or not, followed by methyl methanethiosulfonate (MMTS) treatment. Using this approach, the relative amounts of cysteines that were complexed in a disulfide bridge, i.e., that did not get modified by MMTS in the nonreduced form, could be estimated. The table in Fig. 1C shows the amino acids that were covered (black) or not covered (gray) in the MS bottom-up analysis. As shown in the graphs at the bottom of Fig. 1C, all eBDs showed a clear increase in oxidized cysteines. Especially in the oxidized eBD2 mixture, no cysteines were modified by MMTS; i.e., they were unbound following oxidation. In addition, two precursor masses could be found in the oxidized eBD2 mixture with a charge state above 4+ that disappeared following reduction: 4,327.039 Da (*m/z* of 866.416 as 5+ and *m/z* of 1,082.767 as 4+) and 4,256.000 Da (*m/z* of 852.207 as 5+) (Fig. 1D). The first is the calculated mass of the amino acid sequence (4,336.12 Da) minus 6 Da; i.e., three disulfide bridges formed. The latter is this sequence without the initial alanine (71.037 Da). This residual undigested precursor can be expected, based on the fact that the folded form is less accessible to trypsin, resulting in a top-down MS signal.

### Distribution of eBDs across the equine respiratory tract.

**FIG 2:**
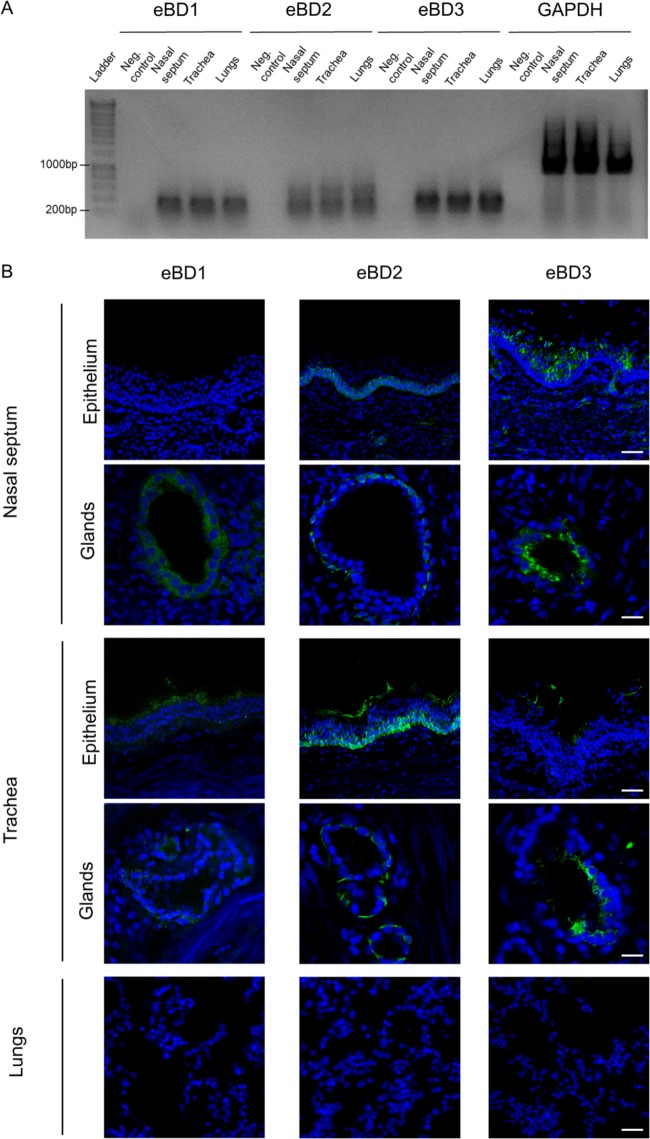
Distribution of eBD1, -2, and -3 across the equine respiratory tract. (A) mRNA expression of eBD1, -2, and -3 in the equine nasal septum, trachea, and lungs. (B) Representative confocal images of eBD1, -2, and -3 protein expression (green) in the equine nasal septum, trachea, and lungs. Cell nuclei were counterstained with Hoechst (blue). Bars, 50 μm.

The expression and localization of eBD1, -2, and -3 in the horses’ respiratory tract were analyzed by means of reverse transcriptase PCR (RT-PCR) and immunofluorescence staining. As shown in Fig. 2A, RNA specific for eBD1, -2, and -3 was expressed throughout the three major parts of the respiratory tract of all five horses (i.e., the nasal septum, the trachea, and the lungs). RT-PCR products were Sanger sequenced, and identities were confirmed through comparison with the respective sequences, published in the NCBI database. eBD1, -2, and -3 protein expression was detected in the nasal septum and trachea but not in the lungs of all five horses (Fig. 2B). More specifically, eBD1 was located within the cytoplasm of the nasal septum’s secretory gland cells but was rarely found in the epithelial cells lining the luminal surface. On the contrary, in tracheal tissues, eBD1 was clearly visible in the cytoplasm of surface epithelial cells, especially at their apical side, as well as in that of the glandular cells. eBD2 was mainly expressed in the basal layers of both the surface epithelium and the glands of the nasal septum and of the trachea. In addition, eBD2 appeared as a secreted smear on top of the surface epithelia. eBD3 displayed a heterogeneous distribution pattern throughout the surface epithelium, and an intense cytoplasmic immunopositive staining was observed within the glandular cells.

### While eBD2 and -3 are potent antimicrobial peptides, they enhance herpesviral infectivity. (i) eBD2 and -3 but not eBD1 inhibits equine bacterial pathogens.

To ensure that eBD1, -2, and -3 are potent antimicrobial peptides, a MIC test was performed to determine inhibition of the growth of a number of Gram-positive and Gram-negative bacteria found in the equine respiratory tract by eBDs. The examined concentration range of eBDs (0.1 ng to 100 μg/ml) was chosen based on the antimicrobially active concentration range of human BDs (21–25).

The minimal concentrations of eBD1, -2, and -3 inhibiting bacterial growth ranged from 25 to 100 μg/ml and are represented in Table 2. While eBD1 (100 μg/ml) showed no antibacterial activity and eBD2 solely inhibited the growth of the Gram-negative bacteria, eBD3 exhibited a broad-spectrum activity against both Gram-negative and Gram-positive bacteria.

**TABLE 2:** Antibacterial activity of eBD1, -2, and -3^*a*^

Bacterium	Gram-staining reaction	MIC (μg/ml)		
		eBD1	eBD2	eBD3
Streptococcus equi subsp. *equi* (3830)	+	>100	>100	100
Streptococcus equi subsp. *zooepidemicus* (4001)	+	>100	>100	>100
Staphylococcus aureus (3939)	+	>100	>100	>100
Rhodococcus equi (3851)	+	>100	>100	25
Actinobacillus equuli subsp. e*quuli* (4005)	−	>100	25	25
Bordetella bronchiseptica (3033)	−	>100	100	100

a The activity of eBD1, -2, and -3 on the growth of 4 Gram-positive and 2 Gram-negative equine bacterial isolates commonly associated with respiratory disease was tested. Bacteria were cocultured with 0 to 100 μg/ml eBD1, -2, or -3 for 24 h, and MIC values were determined to be the lowest concentration of eBD at which there was no visible bacterial growth.

### (ii) eBD2 and -3 inhibit two enveloped viruses but enhance EHV1 infectivity.

Two major respiratory enveloped viruses (equine influenza virus [EIV] and equine arteritis virus [EAV]) were compared to the enveloped herpesvirus EHV1 in their susceptibility to eBD1, -2, and -3 during different steps in the infection of rabbit kidney (RK13) cells (by EAV and EHV1), Madin-Darby canine kidney (MDCK) cells (by EIV), or equine respiratory epithelial cells (EREC) (by EIV and EHV1).

**FIG 3:**
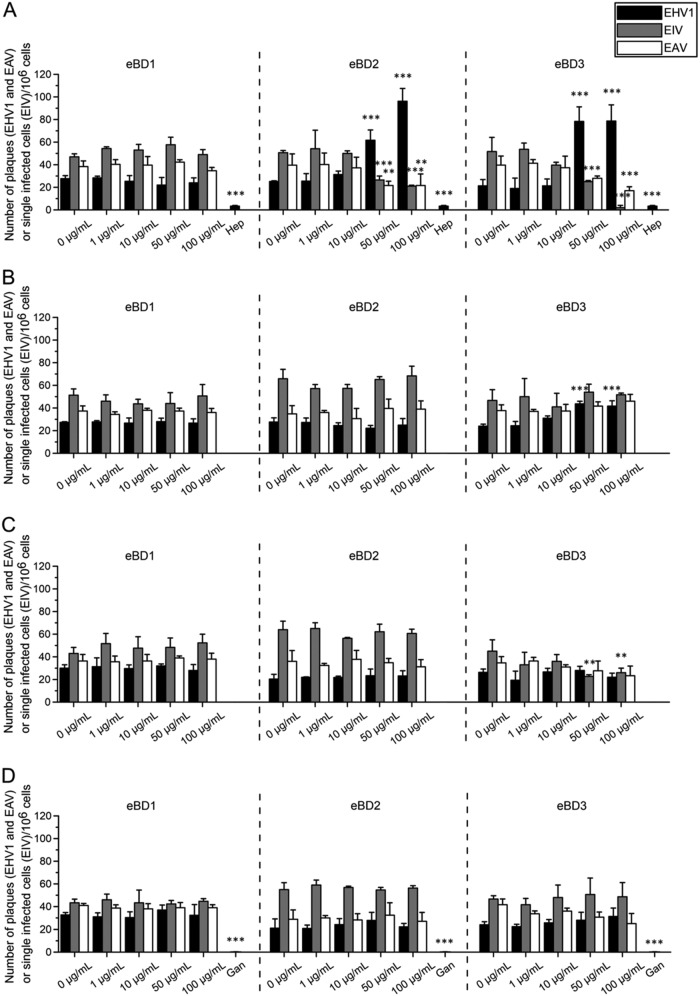
Antiviral activity of eBD1, -2, and -3 during a virus plaque assay on continuous cell lines. The impact of eBD1 (left), eBD2 (middle), and eBD3 (right) was tested on different steps in the infection of continuous cell lines (RK13 cells for EHV1 and EVA or MDCK cells for EIV) with EHV1 (MOI, 0.001), EIV (MOI, 0.001), and EVA (MOI, 0.001). Experiments were performed in triplicate. Significant differences are indicated by asterisks: **, *P* < 0.01; ***, *P* < 0.001. (A) Virus particles were pretreated with 0 to 100 μg/ml eBD1, -2, and -3 prior to inoculation for 2 h at 4°C. Heparin (Hep; 100 U/ml) was used as a positive internal control for blocking subsequent EHV1 binding. (B) Cells were pretreated with 0 to 100 μg/ml eBD1, -2, and -3 prior to virus inoculation for 2 h at 4°C. (C) eBD1, -2, and -3 (0 to 100 μg/ml) were added during the virus entry step for 1 h at 37°C. (D) Following citrate treatment (pH 3), 0 to 100 μg/ml eBD1, -2, and -3 was added to the inoculated cells during the virus postpenetration step for 48 h. Ganciclovir (Gan; 10 μg/ml) was used as a positive internal control to block EHV1 replication.

**FIG 4:**
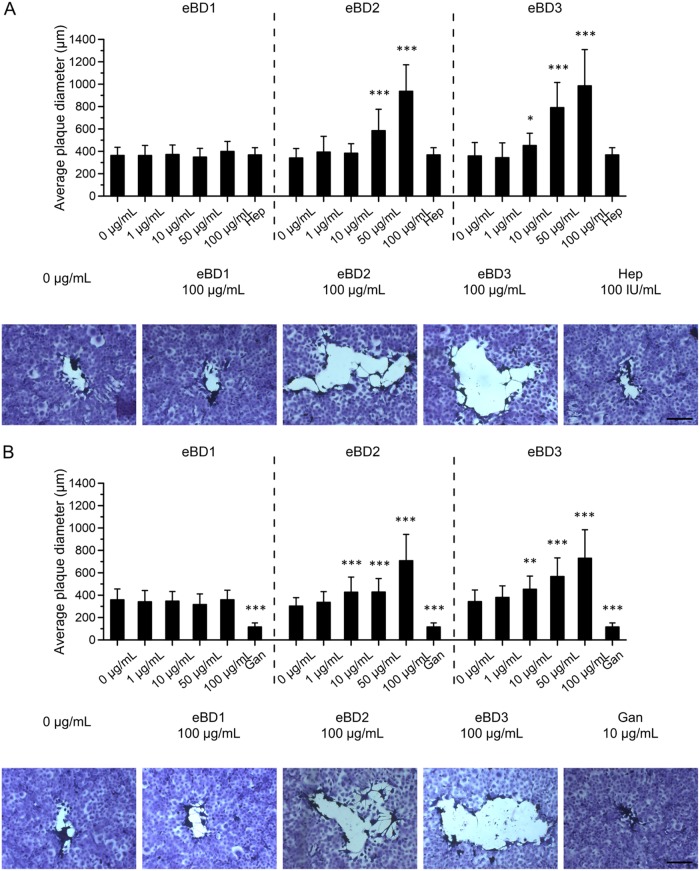
Effect of eBD1, -2, and -3 on EHV1 spread in RK13 cells. eBD1 (top left), eBD2 (top middle), or eBD3 (top right) was added at 0 to 100 μg/ml to the virus in the virus pretreatment step (A) or to EHV1-inoculated RK13 cells during the virus postpenetration step (B). Data from three independent experiments are represented as means + SD (top). Significant differences are indicated by asterisks: *, *P* ≤ 0.05; **, *P* < 0.01; ***, *P* < 0.001. Heparin (Hep; 100 U/ml) was used as a positive internal control during virus pretreatment for blocking subsequent EHV1 binding. Ganciclovir (Gan; 10 g/ml) was used as a positive internal control to block EHV1 replication. Representative images of EHV1 plaques in crystal violet-stained RK13 cell monolayers in the bottom panels of A and B. Bars, 200 μm.

*(a) RK13 and MCDK cells.* (i) As shown in Fig. 3A, virus pretreatment with 50 or 100 μg/ml eBD2 and -3 but not with eBD1 significantly (*P* < 0.01 and *P* < 0.001, respectively) decreased the formation of EIV and EAV plaques in MDCK and RK13 cells, respectively, compared to the plaque formation achieved with 0 μg/ml. eBD3 was particularly active against EIV, almost completely preventing infection at a concentration of 100 μg/ml. Remarkably, the same eBD2 and -3 concentrations (50 or 100 μg/ml) caused an average 3.5-fold increase (*P* < 0.001) in EHV1 infection in RK13 cells, observed both in the number of plaques and in the plaque diameter (Fig. 3A and Fig. 4A, top, respectively). As expected, heparin pretreatment of EHV1 hindered the virus from subsequently binding to RK13 cells and significantly (*P* < 0.001) inhibited EHV1 plaque formation but did not alter the final plaque diameter. Representative images of EHV1 plaques are shown in Fig. 4A, bottom. (ii) Pretreatment of the cells with eBD1, -2, and -3 failed to protect them from subsequent EIV, EAV, or EHV1 infection. In contrast, pretreatment of RK13 cells with 50 or 100 μg/ml eBD3 significantly (*P* < 0.001) increased their susceptibility to EHV1 (number of plaques) by 1.5-fold (Fig. 3B). Cell pretreatment with eBDs did not alter subsequent EHV1 plaque spread (data not shown). (iii) Supplementation of the medium with 50 or 100 μg/ml eBD3 during the virus entry step significantly (*P* < 0.01) decreased EIV infectivity but had no effect on EAV or EHV1 infectivity (Fig. 3C). In addition, the EHV1 plaque diameter was not affected by treating the RK13 cells with eBDs during virus entry (data not shown). (iv) As shown in Fig. 3D, all eBDs failed to alter the number of plaques post-virus penetration. However, the presence of eBD2 and -3 allowed EHV1 to spread faster to neighboring cells in a dose-dependent manner, doubling the average viral plaque diameter at 100 μg/ml compared to that for the control (*P* < 0.001) (Fig. 4B, top). As expected, addition of ganciclovir to RK13 cells almost completely blocked the formation and spread of EHV1 plaques (*P* < 0.001). Representative images of EHV1 plaques are shown in Fig. 4B, bottom. Over the course of the experiment, the EAV plaque diameter was never affected (data not shown).

**FIG 5:**
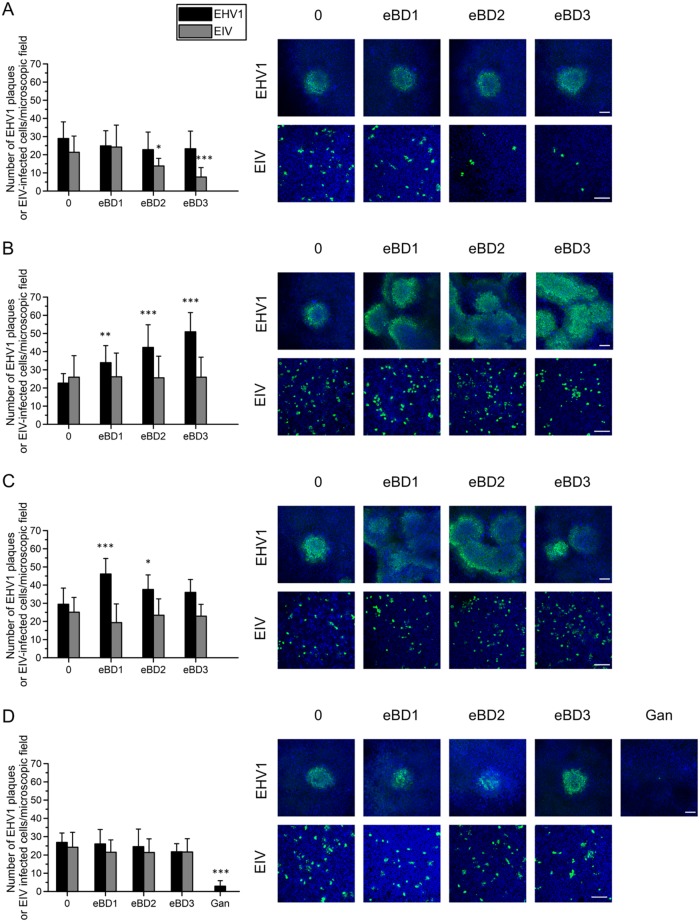
Antiviral activity of eBD1, -2, and -3 during a virus plaque assay on primary EREC. (A) Virus particles were pretreated with 0 or 100 μg/ml eBD1, -2, and -3 prior to inoculation for 2 h at 4°C. (B) Cells were pretreated with 0 or 100 μg/ml eBD1, -2, and -3 prior to virus inoculation for 2 h at 4°C. (C) eBD1, -2, and -3 (0 or 100 μg/ml) were added during the virus entry step for 1 h at 37°C. (D) Following citrate treatment (pH 3), 0 or 100 μg/ml eBD1, -2, and -3 was added to the inoculated cells during the virus postpenetration step for 48 h. Ganciclovir (Gan; 10 μg/ml) was used as a positive internal control to block EHV1 replication. Experiments were performed in triplicate on primary EREC from three different horses. Data are represented as means + SD, and significant differences are indicated by asterisks: *, *P* ≤ 005; **, *P* < 0.01; ***, *P* < 0.001. Representative confocal images of EHV1-positive plaques or EIV-positive individual cells (green) are shown on the right for each step. Cell nuclei were counterstained with Hoechst (blue). Bars, 75 μm.

*(b) EREC.* EREC were grown in transwells to full differentiation, as assessed by measuring the transepithelial electrical resistance (26). Since EHV1 preferentially infects EREC at their basolateral surfaces and EIV more efficiently infects EREC at their apical surfaces, cells were inoculated with EHV1 or EIV via the respective preferential route (26). EAV does not readily infect EREC and was therefore excluded from this experiment. (i) In accordance with what was observed in MDCK cells, eBD2 and -3 acted on EIV virions during pretreatment, significantly (*P* < 0.05 and *P* < 0.001, respectively) diminishing subsequent infection of EREC (Fig. 5A). While eBD2- and eBD3-pretreated EHV1 virions infected RK13 cells more efficiently than nonpretreated virions, they were not able to infect EREC more efficiently. (ii) Pretreatment of EREC with eBD1, -2, and -3 did not alter EIV infectivity, while pretreatment rendered the cells more susceptible to EHV1 infection, as demonstrated by the significant increase in the number of plaques (*P* < 0.01 for eBD1 and *P* < 0.001 for eBD2 and -3). The results are shown in Fig. 5B. (iii) As shown in Fig. 5C, addition of the eBDs during virus entry enhanced subsequent EHV1 infection in EREC (*P* < 0.001 for eBD1, *P* < 0.05 for eBD2, and *P* = 0.1 for eBD3), while it had no effect on EIV entry. (iv) None of the eBDs affected viral infectivity after EHV1 or EIV entry was completed (Fig. 5D). As expected, addition of ganciclovir to EREC almost completely blocked the formation and spread of EHV1 plaques (*P* < 0.001). Representative confocal images of EHV1- and EIV-infected EREC are shown in the right panels of Fig. 5. EHV1 plaque diameter did not significantly change upon addition of eBDs in any of the examined viral infection steps (data not shown).

### Envelope glycoprotein gM renders EHV1 resistant to eBD2, while virion surface glycans are involved in the eBD-induced increase in EHV1 infection.

**FIG 6:**
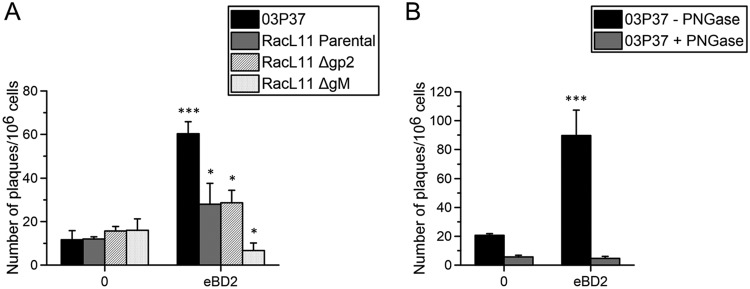
Role of glycoprotein M and virion surface glycans in EHV1 resistance against eBD2 and the eBD2-induced increase in EHV1 infection, respectively. (A) The EHV1 03P37 strain, the parental RacL11 strain, the RacL11 strain lacking glycoprotein 2 (gp2), and the RacL11 strain lacking glycoprotein M (gM) were pretreated with 0 or 100 μg/ml eBD2 prior to inoculation of RK13 cell monolayers. (B) The EHV1 03P37 strain was pretreated or not pretreated with PNGase F (25,000 U/ml) overnight prior to treatment with eBD2 and inoculation of RK13 cell monolayers. Experiments were performed in triplicate, and significant differences are indicated by asterisks: *, *P* ≤ 0.05; ***, *P* < 0.001.

Glycoprotein M (gM) is a type III integral membrane protein conserved across the entire herpesvirus family. Since gM spans the viral envelope multiple times, we hypothesized that it stabilizes and protects the viral envelope from damage by membrane-penetrating eBDs. Previous experiments were performed with a recent field strain, strain 03P37, isolated from the blood of a paralytic horse (27). For this experiment, we used the older laboratory-adapted RacL11 strain, of which several mutants have been constructed, including a RacL11 ΔgM strain. Similar to the infectivity of the 03P37 strain, the infectivity of the parental RacL11 strain in RK13 cells was enhanced (*P* < 0.05) upon pretreatment with 100 μg/ml eBD2, although to a lesser extent. In contrast, the infectivity of the RacL11 strain deficient in gM (RacL11 ΔgM) was significantly (*P* < 0.05) diminished upon incubation with 100 μg/ml eBD2 compared to that of the control (0 μg/ml), as shown in Fig. 6A. The RacL11 strain deficient in the single membrane-spanning glycoprotein 2 (RacL11 Δgp2) was included as an internal control. The RacL11 Δgp2 strain showed a significant (*P* < 0.05) increase in infectivity similar to that of the parental RacL11 strain upon virion pretreatment with eBD2. These findings suggest that gM might play a role in protecting EHV1 virions from direct eBD2 antiviral effects.

Since human BDs have been shown to exert lectin-like functions, we tested whether viral N-linked glycans are involved in the observed increase in EHV1 infection upon virion pretreatment (25, 28). Following removal of N-linked glycans with the use of peptide-*N*-glycosidase F (PNGase F) on EHV1 surfaces, the proviral effect of the eBDs was diminished (Fig. 6B). These results suggest that virion surface glycans, rather than gM, are involved in the increase in EHV1 infection upon pretreatment with eBD2.

### eBDs enhance EHV1 infectivity by aggregating virions on the cell surface.

A binding assay with purified and DiO-labeled EHV1 particles was done to characterize the attachment of either eBD1-, eBD2-, or eBD3-pretreated EHV1 particles to RK13 cells or untreated EHV1 particles to eBD1-, eBD2-, or eBD3-pretreated EREC.

### (i) RK13 cells.

**FIG 7:**
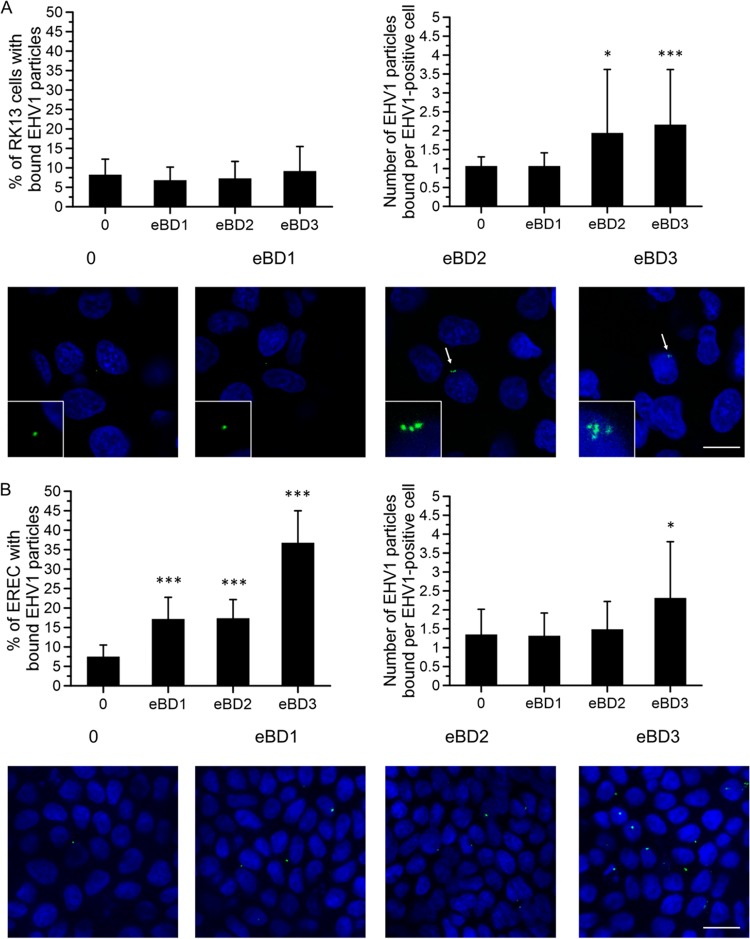
Role of eBD1, -2, and -3 in the attachment of EHV1 to cells. (A) The binding of eBD1, -2, and -3 (0 or 100 μg/ml)-pretreated and DiO-labeled EHV1 particles to RK13 cells. (B) The attachment of untreated and DiO-labeled EHV1 particles to eBD1, -2, and -3 (0 or 100 μg/ml)-pretreated EREC. (Top left) The percentage of cells with bound virus. (Top right) The total number of particles counted per EHV1-positive cell. Three independent experiments were performed with RK13 cells, and EREC from three different horses were used. Data are represented as means + SD, and significant differences are indicated by asterisks: *, *P* ≤ 0.05; ***, *P* < 0.001. (Bottom) Representative confocal z-stack images of eBD1, -2, and -3-pretreated and DiO-labeled EHV1 particles (green) attached to RK13 cells (A) or untreated and DiO-labeled EHV1 particles (green) bound to eBD1, -2, and -3-pretreated EREC (B). Note the virus aggregates (arrows) bound to the RK13 cells upon virus pretreatment with eBD2 and -3. Magnified (×4) images are shown in the lower left corner of each picture. Cell nuclei were counterstained with Hoechst (blue). Bars, 10 μm.

As shown at the top of Fig. 7A, the percentage of RK13 cells with bound EHV1 particles was slightly, but not significantly, higher after virus pretreatment with 100 μg/ml eBD3 (1.26% ± 1.34%) than after virus pretreatment with eBD1 (100 μg/ml; 0.78% ± 0.76%) or eBD2 (100 μg/ml; 0.86% ± 0.97%) or after the control (0 μg/ml; 0.92% ± 0.92%) pretreatment. However, per positive cell, up to 2-fold more virus particles were able to bind to the cell surfaces upon treatment with eBD2 and -3 than upon treatment with eBD1 or upon the control pretreatment. As shown in the confocal images at the bottom of Fig. 7A, eBD2- and eBD3-pretreated EHV1 particles were concentrated to one specific area of the cell membrane.

### (ii) EREC.

Pretreatment of EREC with all eBDs (100 μg/ml) significantly increased the percentage of cells with bound EHV1 particles from 7% ± 3% to 17% ± 6% (eBD1), 17% ± 5% (eBD2), and 36% ± 8% (eBD3) (Fig. 7B, top). The number of EHV1 particles bound per EHV1-positive cell did not significantly differ between control and eBD1- and eBD2-treated EREC. eBD3 pretreatment of EREC doubled the amount of bound EHV1 particles per positive cell. Representative confocal images are shown at the bottom of Fig. 7B.

### EHV1 specifically induces eBD expression in EREC.

**FIG 8:**
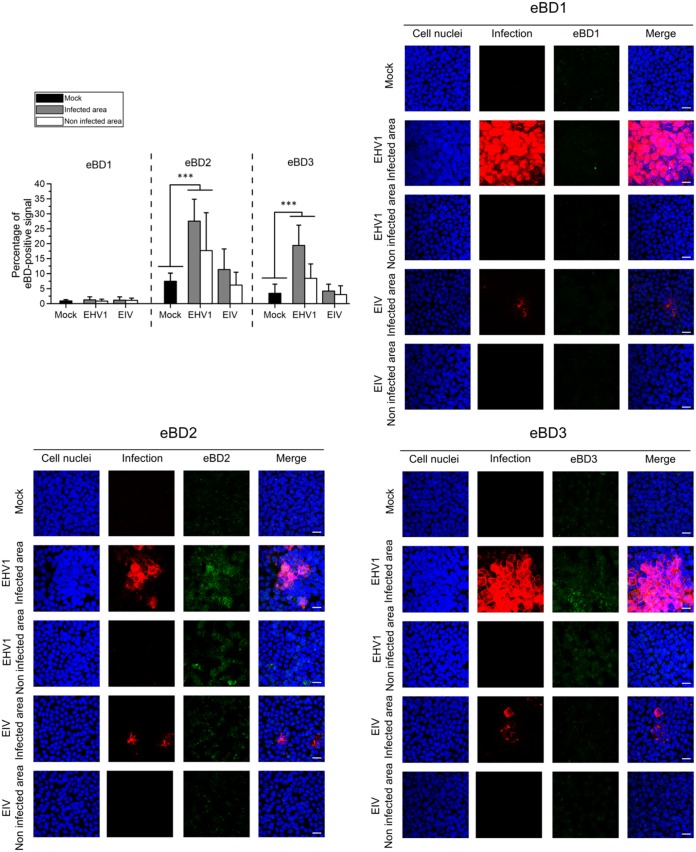
Viral infections elicit eBD2 and -3 protein expression in EREC. Cells were either mock inoculated or inoculated with EHV1 (MOI, 0.2) or EIV (MOI, 2) for 18 h. (Top left) The mean percentage of the eBD1 (left), eBD2 (middle), or eBD3 (right) fluorescent signal was calculated on 5 complete z-stack confocal images using ImageJ software. The mean fluorescent signal of the mock-inoculated cells (black bars) was compared to that of EHV1- or EIV-inoculated cells, with the last two groups containing infected as well as noninfected areas. Experiments were performed in triplicate on primary EREC from three different horses. Data are represented as means + SD, and significant differences are indicated by asterisks: ***, *P* < 0.001. (Top right and bottom) Representative confocal z-stack images of eBD1, -2, and -3 protein expression in mock-, EHV1-, or EIV-inoculated EREC, with the last two groups containing infected as well as noninfected areas. eBD1, -2, and -3 were visualized in green, while EHV1 late proteins and EIV nucleoprotein were simultaneously stained in red to distinguish infected from noninfected areas. Cell nuclei were counterstained with Hoechst (blue). Bars, 10 μm.

We determined if infection affected eBD expression at their target site in EREC. As shown in the graph and representative confocal images in Fig. 8, EREC constitutively expressed a small amount of eBD1, which did not significantly differ among mock-inoculated cells, EHV1-inoculated cells, and EIV-inoculated cells. However, the mean percentage of the eBD2-positive signal was significantly (*P* < 0.001) higher in EHV1-inoculated cells than in mock-inoculated cells. Infected cells within the EHV1-inoculated EREC monolayers showed a high percentage of the eBD2-positive signal (27.54% ± 7.32%), while their noninfected neighboring cells expressed an eBD2-positive signal of only 17.68% ± 12.67%. EIV-inoculated cells displayed a slight, but not significant, increase in eBD2 expression. Among EIV-inoculated cells, infected cells did show statistically significantly higher eBD2-positive signals (11.33% ± 6.90%) than neighboring noninfected cells (6.21% ± 4.22%). Similar to the findings for eBD2, eBD3 expression was significantly (*P* < 0.001) elevated in EHV1-inoculated cells compared to that in mock-inoculated cells. Again, EHV1-infected cells expressed, on average, more eBD3 (19.40% ± 6.79%) than adjacent noninfected cells (8.47% ± 4.74%). EIV inoculation did not lead to a significant increase in eBD3 expression in EREC compared to that achieved with mock inoculation.

### eBDs are mildly chemotactic for equine leukocytes.

**FIG 9:**
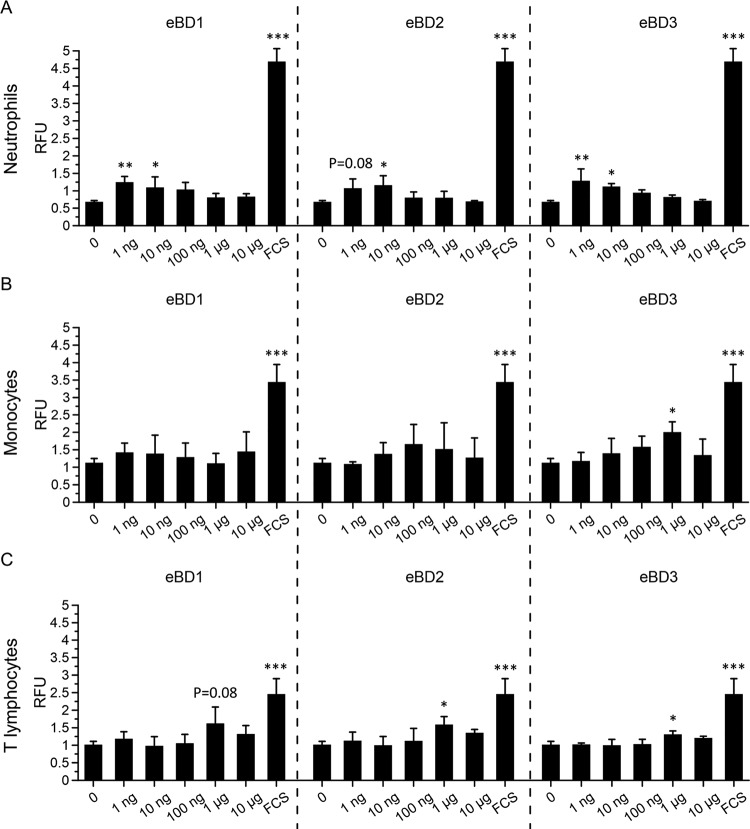
Chemotactic activity of eBD1, -2, and -3 on equine blood leukocytes. Migration of neutrophils (A), CD172a^+^ monocytes (B), and CD3^+^ T lymphocytes (C) induced by 0 to 10 μg/ml eBD1 (left), eBD2 (middle), or eBD3 (right). Fetal calf serum (FCS) was used as an internal positive control. Experiments were performed in triplicate on blood leukocyte subpopulations from three different healthy horse donors. Cell migration values are reported as relative fluorescence units (RFU) and represented as means + SD. Significant differences are indicated by asterisks: *, *P* ≤ 0.05; **, *P* < 0.01; ***, *P* < 0.001.

We next examined the effect of the increased expression of eBD at sites of infection on leukocyte chemotaxis. CytoSelect (Cell Biolabs, San Diego, CA, USA) cell migration assays were performed with equine polymorphonuclear (PMN) cells, CD172a^+^ mononuclear cells, and CD3^+^ T lymphocytes isolated from the blood taken from three different healthy donor horses. The results are shown in Fig. 9 and reveal a dose-dependent migration of these blood leukocytes toward the different eBDs in a typical Gaussian manner. More specifically, eBD1 induced a mild and dose-dependent migration of equine PMN cells and of equine T lymphocytes, with maximal migration occurring at 1 ng/ml (*P* < 0.01) and 1 μg/ml (*P* = 0.08), respectively. The chemotactic effect of eBD2 peaked between 1 ng/ml (*P* = 0.08) and 10 ng/ml (*P* < 0.05) for equine PMN cells and at 1 μg/ml (*P* < 0.05) for T lymphocytes. eBD3 induced the migration of equine PMN cells, monocytic cells, and T lymphocytes, reaching a maximum at a concentration of 1 ng/ml (*P* < 0.01), 1 μg/ml (*P* < 0.05), and 1 μg/ml (*P* < 0.05), respectively. While eBD1 and -2 failed to induce the chemotaxis of equine monocytic cells, 1 μg/ml of eBD3 was sufficient to induce mild monocytic cell migration. RPMI supplemented with 10% fetal calf serum (FCS) served as a positive control and efficiently (*P* < 0.001) induced the chemotaxis of all cell types examined.

## DISCUSSION

Herpesviruses, like all viruses, must modulate, evade, or bypass their hosts defenses in order to replicate and be transmitted to new hosts. β-Defensins are significant antimicrobial peptides in the respiratory tract. Our study aimed at uncovering how alphaherpesvirus virions evade β-defensin action during infection of the host respiratory tract.

The three major equine β-defensins (eBDs) were expressed throughout the major parts of the horses’ upper respiratory tract. There was a marked difference in eBD1 expression between equine nasal and tracheal epithelial cells. eBD1 was low to absent in the former and present in the latter. Since the nasal cavities filter out most of the inhaled debris, microbial invasion might silence eBD1 production in the luminal epithelial cells. In this context, Haemophilus influenzae is known to reduce human BD1 release in bronchial epithelial cells (29). Since the expression of human BD2 and -3 is regulated in a manner distinct from that of human BD1, it is not surprising that eBD1 expression and induction differed from those of eBD2 and -3 (30, 31). The distribution patterns of eBD2 and -3 were similar between nasal and tracheal respiratory epithelia and were similar to previously reported expression profiles in humans (21, 22, 30–32).

Mammalian BDs inactivate bacterial and enveloped viral microorganisms by disrupting the microbial membrane (21–23, 28). Here, we investigated the antimicrobial properties of the three major eBDs. Similar to its human homolog, oxidized eBD1 exhibited minimal antibacterial or antiviral effects (11, 24, 25, 31–33). However, Schroeder et al. in 2011 (23) found that the human BD1 antimicrobial killing activity is revealed under reducing conditions (e.g., in the intestines). Human BD1 might even form bacterium-entrapping nets under these circumstances (34). Unfortunately, the concentrations of DTT necessary for such reducing environments (i.e., the conversion of resazurin to resorufin) affected bacterial and mammalian cell growth, making it impossible to examine the role of reduced eBD1 (data not shown).

Oxidized eBD2 and eBD3 efficiently inhibited several equine bacterial and viral pathogens, primarily through direct actions and at concentrations corresponding to those reported for human BD2 and -3 (21, 22, 24, 25). So far, the exact physiological concentrations of eBDs in equine tissues and body fluids are unknown. Future studies should determine these concentrations and link them with data known from human samples. For instance, human BD concentrations in human vaginal and respiratory lavage fluids are roughly 1,000-fold lower than their active concentrations. However, BDs might have been diluted in these samples, as another study found concentrations up to several micrograms per milliliter in human breast milk and mammary gland epithelia (35). These data suggest that BDs might be present at high local concentrations upon their release from specific cells and can become diluted in extracellular fluids (28).

Gram-negative bacteria generally were more sensitive to eBDs than Gram-positive bacteria. In contrast to Gram-positive bacteria, which are protected by an outer peptidoglycan layer, Gram-negative bacteria have an anionic lipopolysaccharide (LPS) exterior cell membrane. Due to their polycationic and amphipathic nature, defensins can efficiently interact with and subsequently destroy this lipid bilayer (7, 14, 15). Whether the direct inactivation of EIV and EVA by eBD2 and eBD3 was a consequence of direct viral envelope penetration or blocking/cross-linking of viral binding/entry proteins is not clear. Both antiviral mechanisms have been described for defensins acting on a number of viruses, including respiratory syncytial virus, influenza virus, and human immunodeficiency virus (HIV) (11–13).

Herpesvirus virions were not damaged by the markedly antimicrobial eBD2 and -3, due to the incorporation of glycoprotein M (36, 37). These results complement those of an electron microscopy study by Daher et al. performed in 1986 (38), showing that the morphology of the closely related human herpes simplex 1 (HSV1) envelope was not altered by incubation with human α-defensin 1. We propose that the multiple transmembrane-spanning domains of gM protect the virus from lipid bilayer-attacking eBDs by stabilizing the viral envelope. Further evidence of the role of gM in membrane stabilization comes from the fact that the gM glycoproteins of pseudorabies virus and EHV1 inhibit the fusion induced by artificially optimized viral proteins gB and gD (39).

Interestingly, our study revealed that EHV1 evolved to repurpose BDs to increase virion infectivity through both virus- and cell-mediated mechanisms. For the virus-mediated mode of action, our results indicate that N-linked glycans on virion surface glycoproteins participate in the eBD interaction and the subsequent enhancement of infection. In addition, we propose that the cationic eBD2 and -3 peptides electrostatically bind to the viral envelope and/or anionic surface glycoproteins. This binding might enable the formation of virion aggregates, which in turn could increase the change of successful infection. A similar effect has been shown for α-defensins on HIV and murine adenovirus 2 (MAdV-2) (40, 41). In addition, a recent study showed that human BD2 and -3 and human α-defensins 5 and 6 facilitated the binding of HIV and/or human adenovirus (HAdV) to epithelial cells. However, viral infectivity was reduced through the cointernalization of virus particles and defensins, which led to internal virion neutralization (42, 43). Nevertheless, neutralizing the net negative virion charge and lectin-glycan interactions may promote subsequent virion aggregation. For example, infection by HAdV can be enhanced by coating the virions with polycations (44). Due to the inherent ability of eBD2 and -3 to oligomerize, these virion aggregates could potentially expand even further. In line with our observations, human α-defensins aggregate virions of influenza virus, HAdV, and BK virus (11, 45). Plausible eBD2 and -3 target candidates on the surface of EHV1 are the highly glycosylated gC and gB. With the use of surface plasmon resonance, Hazrati et al. (24) showed that human BD3 could bind to gB of HSV1 and -2. However, instead of increasing subsequent HSV infectivity, human BD3 binding to gB inhibited subsequent HSV infectivity in human cervical epithelial cells. However, EHV1 gB is presumably not (solely) involved in eBD binding, as eBD2 was still able to enhance the infection of gB-free EHV1 virions in RK13 cells (data not shown). Similar results were obtained with gC-null EHV1 virions. Interestingly, the 03P37 strain exhibits a point mutation in the gene encoding glycoprotein C, translating an uncharged amino acid at position 166 (glutamine). In contrast, the older RacL11 strain harbors a positively charged amino acid at the latter position (lysine). Although highly speculative, this higher net positive charge in the RacL11 strain might repulse the positively charged eBDs. In turn, eBD pretreatment of RacL11 virions would result in a less clear enhancement of infection compared to the level of infection with the 03P37 strain. Future studies will elucidate the role of the latter amino acid in gC of the 03P37 strain.

The fact that we could not observe an increase in EHV1 infection upon inoculation of EREC could be attributed to the pore size of the transwells. EREC were grown on 400-nm-pore size membranes. As EHV1 particles measure, on average, 150 nm in diameter, virion aggregates may not efficiently reach the basolateral surface of these cells. Membranes with larger pores did not support cell growth, and apical inoculation of the cells did not yield a sufficient infection to test the effect of eBDs (26).

A potential cell-mediated mechanism was noticed after pretreatment of EREC with eBD1, -2, and -3. In this context, eBDs might act at the cell surface in a way that promotes increased infection. We did not observe virion aggregates but did observe an increase in the number of EREC with bound single EHV1 particles. Since a similar proviral effect was observed when eBD1, -2, and -3 were added during the viral entry step, we suggest that eBD1, -2, and -3 bind to an EHV1 binding/entry receptor(s) on the cell surface and thereby facilitate subsequent EHV1 particle binding to and subsequent entry into the cell. eBD1, -2, and -3 might facilitate direct viral binding through neutralizing the net negative charge between the virion and cellular glycoproteins. Alternatively, by inducing a conformation change in EHV1’s binding/entry receptor(s), subsequent EHV1 particle binding and entry might be enhanced. It is not surprising that eBD3 was the most potent peptide out of all three eBDs, considering the fact that eBD3, followed by eBD2, has the highest content of cationic amino acids, which are clustered as a functional unit near the C terminus of the peptide. The effect of eBDs on EHV1 infection during cell pretreatment of RK13 cells was strongly attenuated, which is not surprising, because the RK13 cell line may not recognize foreign eBDs, while EREC are the natural target cells of eBDs.

We observed that in RK13 cells, but not in EREC, cointernalization of these virion aggregates resulted in rapid cell-to-cell spread in the cell monolayer. Since defensins have been shown to promote the fusion of membranes, these defensin-virion aggregates may fuse cells to form syncytia (46). A similar enhancement of viral spread in RK13 cells was observed when eBDs were added after virions had entered the cell. In EREC grown on transwells, EHV1 does not induce syncytia, supporting our hypothesis that eBDs facilitate membrane fusion and subsequent syncytium formation.

Finally, we observed that EHV1 infection of EREC elicited an increase in the levels of eBD2 and -3 in EREC compared to those obtained with EIV infection. Future studies aim to determine the exact concentrations present in the supernatants and lysates of these infected EREC. In addition, these eBDs attracted monocytic cells and T lymphocytes to a mild extent. These cells are essential for EHV1 dissemination and are sites of persistent infection (47, 48). Human BD1 and -2 and mouse BD2 also induce a well-controlled chemotaxis of leukocytes (16, 17). The controlled chemotaxis and inflammation at the site of viral infection may be important for EHV1 survival.

In this report, we demonstrated that the horse’s respiratory tract constitutively produces eBD1, -2, and -3, thereby potentially shaping the microbiota of the upper respiratory tract and protecting the lower respiratory tract from pathogenic invasion. EHV1, a virus that has evolved with its host over long periods of time and that is exquisitely well adapted to it, is resistant to antimicrobial eBDs, presumably due to incorporation of the virus-encoded, multiple transmembrane-spanning gM glycoprotein in the virion envelope. Moreover, EHV1 has evolved to repurpose these eBDs for enhancement of infectivity and dissemination and persistence in its host.

## MATERIALS AND METHODS

### Ethics statement.

The collection of blood from horses was performed according to an institutionally approved protocol adhering to national (Belgian Law 14/08/1986 and Belgian Royal Decree 06/04/2010) and European (EU Directive 2010/63/EU) animal regulations. The protocol was reviewed and approved by the Ethical Committee of Ghent University, Faculty of Veterinary Medicine (EC2017/118). In the abattoir, respiratory tissues were collected from horses that were being processed as part of the normal work of the abattoir. Consent to take the samples was received from the abattoir.

Cryopreserved human respiratory tissues were obtained from a previous *ex vivo* study that was approved by the Ethical Committee of Ghent University Hospital (5). All recruited human participants were of adult age and provided written consent.

### Peptide synthesis, purification, oxidative folding, and validation.

eBD1, eBD2, and eBD3 were chemically synthesized via in-house techniques at Biomatik (Cambridge, Canada) and subsequently purified to >95% by high-performance liquid chromatography (HPLC). The eBD molecular weights were verified by electrospray ionization mass spectrometry (ESI-MS). In the final step, toxic trifluoroacetic acid (TFA), essential during peptide synthesis, was exchanged for nontoxic HCl salt. All eBDs were stored as a lyophilized powder at −20°C until further processing. Lyophilized eBD2 and -3 were thawed and dissolved in 10 mM phosphate buffer (PB; pH 7.7) at a concentration of 0.5 mg/ml. The folding reaction occurred through air oxidation in open vessels, which were gently shaken overnight at room temperature. The oxidation of eBD1 was performed in 20% dimethyl sulfoxide (DMSO) diluted in 0.1 M Tris buffer, pH 6, for 2 h at room temperature, essentially as described by Tam et al. (49). Solutions were finally filtered sterile through 0.22-μm-pore-size filters. Oxidized eBDs were validated using mass spectrometry-based proteomics.

Lyophilized, synthetic peptides of human BD1 and -2 (Alpha Diagnostic International, TX, USA) were reconstituted in ultrapure distilled water (Invitrogen), while lyophilized, recombinant hBD3 expressed in Escherichia coli (Sigma-Aldrich) was reconstituted in 10 mM acetic acid at a concentration of 1 mg/ml according to the instructions of the manufacturer.

### Mass spectrometry. (i) Protocol.

The work flow used to validate eBD oxidation via LC-MS analysis is graphically shown in Fig. 1C. Bottom-up analysis was performed in triplicate to confirm oxidation of the eBDs and quantify the relative abundance of disulfide bridges in the oxidized versus reduced forms. For this, lyophilized oxidized eBD1, -2, and -3 were dissolved in triethylammonium bicarbonate (TEABC) buffer (Sigma-Aldrich) at a concentration of 50 μg/ml. One part was reduced by adding dithiothreitol (DTT; Sigma-Aldrich) to a final concentration of 1 mM, and another part was left oxidized for 1 h at 60°C. Next, free cysteine (C) molecules were alkylated by adding methyl methanethiosulfonate (MMTS; Sigma-Aldrich) to a final concentration of 10 mM for 10 min at room temperature. Acetonitrile (Sigma-Aldrich) was added to a final concentration of 5% to facilitate mass spectrometry (MS) analysis of the samples. Finally, trypsin (trypsin/protein ratio, 1:20; Promega) and CaCl_2_ at a final concentration of 10 mM were added for overnight digestion at 37°C. The peptides were then identified through LC-ESI-MS. LC was performed using a nanoAcquity ultraperformance liquid chromatography (UPLC) system (Waters, Zellik, Belgium). First, samples were delivered to a trap column (180-μm by 20-mm nanoAcquity UPLC 2G-V/MTrap 5-μm-particle-size Symmetry C_18_ column; Waters) at a flow rate of 8 μl/min for 2 min in 99.5% buffer A (0.1% formic acid). Subsequently, the peptides were transferred to an analytical column (100-μm by 100-mm nanoAcquity UPLC 1.7-μm-particle-size Peptide BEH column; Waters) and separated at a flow rate of 300 nl/min using a gradient of 60 min, going from 1% to 40% buffer B (0.1% formic acid in acetonitrile). MS data acquisition parameters were set as described by Helm et al. (50), with minor adaptations.

### (ii) Data analysis.

Progenesis QI for Proteomics software (Progenesis QIP, version 2.0; Nonlinear Dynamics, Waters) was used to process the raw LC-MS data of the triplicate measurements. Rank 3 MS/MS spectra of the MS precursors were exported as separate *.mgf peaklists. An error-tolerant search against an NCBI Equus FASTA file (downloaded February 2018) supplemented with internal standards and the CRaP database was performed using a Mascot (version 2.5) in-house server (Matrix Science), with methylthio and ammonia loss (KNQR) being variable modifications. Mass error tolerance for the precursor ions was set at 10 ppm, and that for the fragment ions was set at 50 ppm. Enzyme specificity was set to trypsin, allowing for up to two missed cleavages. These results were loaded back into Progenesis software, and the eBD peptides were flagged and exported with their ion intensities. The relative abundance of disulfide bridge formation was assessed by summing all methylthio peptide intensities and dividing them by the total sum of methylthio peptides in the reduced samples, i.e., 100%.

### Gel electrophoresis and Western blotting for antibody validation.

First, Western blotting of synthetic eBD1, -2, and -3 and hBD1, -2, and -3 was performed to ensure the specificity and sensitivity of the anti-human BD (hBD) antibodies for the eBDs, using the respective anti-hBD antibodies. Briefly, 10 μg (BD1) or 3 μg (BD2 and -3) of synthetic peptides was boiled in Tricine sample buffer with β-mercaptoethanol and loaded onto 18% T, 6% C Tricine-SDS polyacrylamide minigels (51, 52). The gels were run at 60 V for 1 h and 80 V for 3 h using a Mini Protean Tetra apparatus (Bio-Rad, Hercules, CA, USA) and transferred at 4°C for 50 min at 100 V onto 0.2-μm-pore-size polyvinylidene difluoride (PVDF) membranes (Bio-Rad) in Towbin buffer. The blot membrane was dried and kept at −20°C. After briefly being soaked in methanol, rinsed in ultrapure water, fixed with 0.01% glutaraldehyde in phosphate-buffered saline (PBS) for 20 min for eBD1 and hBD1, and rinsed in ultrapure water, aspecific binding sites were blocked by incubating the membrane in PBS with 0.01% Tween 20 (PBST) and 5% nonfat milk for 1 h at room temperature. Peptides were immunodetected using the respective polyclonal rabbit anti-human BD1, -2, and -3 antibodies (see below), diluted 1:500 in PBST with 5% milk, followed by incubation of the membrane with a goat anti-rabbit IgG horseradish peroxidase (HRP)-conjugated antibody (Agilent, Santa Clara, CA, USA). The blot was developed with an Amersham ECL Prime detection kit (GE Healthcare Life Sciences, Pittsburgh, PA, USA). Coomassie blue staining of the PVDF membrane was performed to assess remnant eBD1 and hBD1 levels on the blot membrane as previously published (53). Pictures were taken with a ChemiDocMP imaging system (Bio-Rad).

### Tissue collection.

The nasal septa, tracheas, and lungs from 5 different healthy horses were collected at an abattoir and transported in phosphate-buffered saline (PBS) with calcium and magnesium supplemented with antibiotics and amphotericin B (Thermo Fisher Scientific, Waltham, MA, USA). The nasal and tracheal mucosa was stripped from the underlying cartilage and, along with the lung tissues, cut into small square pieces (25 mm^2^). All tissues were snap-frozen and stored at −80°C until further processing.

### Immunofluorescence staining and confocal microscopy.

**(i) Tissue fragments.** Fifteen-micrometer-thick cryosections were cut using a cryostat at −20°C and loaded onto 3-aminopropyltriethoxysilane (Sigma-Aldrich, St. Louis, MO, USA)-coated glass slides. The slides were then fixed in 4% paraformaldehyde (PFA) for 15 min, and the cryosections were subsequently permeabilized in 0.1% Triton X-100 diluted in PBS. Cryosections were stained for eBD1, -2, and -3 using rabbit polyclonal anti-human BD1 (catalog number ab115813; Abcam, Cambridge, UK), anti-human BD2 (catalog number ab183214; Abcam), and anti-human BD3 (catalog number ab19270; Abcam), respectively. The immunogens of the antibodies against human BD1, -2, and -3 had homologies of >75% with the respective equine forms. To ensure the sensitivity of the antibodies for eBDs, electrophoresis and Western blotting of synthetically produced eBD1, -2, and -3 were performed, as described below. Specificity was verified by simultaneously staining human respiratory tissues. A rabbit polyclonal antibody against Streptococcus suis (Emelca Bioscience, Antwerp, Belgium) was used as an isotype control antibody. Antibodies, diluted in PBS with Ca and Mg with 10% negative goat serum (NGS), were used overnight at 4°C, followed by incubation with a goat anti-rabbit IgG fluorescein isothiocyanate (FITC)-conjugated antibody (Thermo Fisher Scientific) for 1 h at 37°C. Nuclei were detected by staining with Hoechst 33342 (Thermo Fisher Scientific). Slides were mounted with glycerol-1,4-diazabicyclo[2.2.2]octane (DABCO), and pictures were taken using a Leica confocal microscope (model TCS SPE; Leica Microsystems, Wetzlar, Germany).

**(ii) EREC.** Immunofluorescence staining to visualize EHV1 late proteins and eBDs was performed directly in the transwells. Antibodies were incubated for 1 h at 37°C, unless stated otherwise. Cells were first incubated with a polyclonal biotinylated horse anti-EHV1 antibody (54) or a mouse monoclonal anti-influenza A virus nucleoprotein antibody (catalog number HB-65; American Type Culture Collection [ATCC]) diluted in PBS with 10% NGS, followed by incubation with streptavidin-FITC (Thermo Fisher Scientific) or a goat anti-mouse IgG FITC-conjugated antibody (Thermo Fisher Scientific), respectively. If indicated, the cells were simultaneously stained for viral proteins and eBDs overnight at 4°C. For this, the transwells were incubated with the latter anti-EHV1 or anti-EIV antibodies, together with the rabbit polyclonal anti-BD1, -2, or -3 or isotype control antibodies. EHV1 and EIV proteins were subsequently visualized with streptavidin-Texas Red (Thermo Fisher Scientific) or a Texas Red-conjugated goat anti-mouse IgG antibody (Thermo Fisher Scientific), while eBDs were detected with a goat anti-rabbit immunoglobulin FITC-conjugated antibody. Nuclei were counterstained with Hoechst 33342 for 10 min at 37°C. The transwell membranes were excised from the culture inserts and mounted on glass slides using glycerol-DABCO. The slides were examined using a Leica confocal microscope.

### RT-PCR.

Reverse transcriptase PCR (RT-PCR) was performed on total RNA extracted from nasal, tracheal, and lung tissue fragments of five different horses. The housekeeping gene *GAPDH* (glyceraldehyde-3-phosphate dehydrogenase) was used as an internal control, and reactions without RNA functioned as negative controls. Primers and predicted cDNA sizes are given in Table 3 and were designed based on published and predicted equine nucleotide sequences from the NCBI database, using the Integrated DNA Technologies (IDT) Primer Design tool, and were commercially synthesized (IDT, Coralville, IA, USA). Briefly, fragments from all tissues were disrupted in RTL lysis buffer, supplied with the RNeasy minikit (Qiagen, Hilden, Germany), and homogenized by passing the lysate several times through a blunt 20-gauge needle. Total RNA was extracted from the lysate using a Qiagen RNeasy minikit (Qiagen), following the manufacturer’s instructions, and treated with 120 U DNase I (New England BioLabs, Ipswich, MA, USA) to eliminate genomic DNA contamination. Reverse transcriptase PCR (RT-PCR) of 1 μg of RNA was performed in a Bio-Rad T100 thermal cycler using a Qiagen one-step RT-PCR kit following the manufacturer’s guidelines with eBD-specific forward and reverse primers. PCR products were run on a 1% agarose gel, subsequently stained with ethidium bromide, and visualized with a Gel Doc 1000 imaging system (Bio-Rad). All PCR products were purified for sequencing using a PCR cleanup gel extraction kit (Macherey-Nagel, Düren, Germany) and sent to GATC Biotech (Constance, Germany) for Sanger sequencing. All sequenced PCR products were confirmed using MEGA software, version 6 (Molecular Evolutionary Genetics Analysis [MEGA], Philadelphia, PA, USA).

**TABLE 3:** Primer design for eBD1, -2, and -3 and GAPDH^*a*^

Gene	Primer sequence (5′-3′)		NCBI accession no.	Predicted size of amplified cDNA (bp)
	Forward	Reverse		
GAPDH	AGGTCGGAGTAAACGGATTTG	CATAAGGTCCACCACCCTATTG	NM_001163856	971
eBD1	CCTCTGGAAGCCTCTGTCA	TTCCCGCCGTAACAAGTGC	XM_005606422	269
eBD2	CTTCCTCATTGTCTTCCTGTT	TAGCAGTTTCTGACTCCACATC	NM_001081887	279
eBD3	TCTTCGCATTGCTCTTTCT	GCTTCTATAAACTTCAAGGAGGCA	XM_003364245	275

a Primers were designed based on published and predicted equine nucleotide sequences from the NCBI database.

### Bacteria and MIC assays.

Clinical bacterial isolates were used in a MIC assay to investigate the sensitivity of bacterial pathogens from the equine respiratory tract to eBDs. The test organisms included the Gram-positive bacteria Streptococcus equi subsp. *zooepidemicus* (strain 4001), Streptococcus equi subsp. *equi* (strain 3830), Staphylococcus aureus (strain 3939), and Rhodococcus equi (strain 3851) and the Gram-negative bacteria Actinobacillus equuli subsp. *equuli* (strain 4005) and Bordetella bronchiseptica (strain 3033). All organisms were grown on Oxoid sheep blood agar plus plates (Thermo Fisher Scientific) overnight in a humidified incubator (5% CO_2_) at 37°C. Inocula were prepared by swabbing bacterial colonies from the plates, suspending them in PBS (turbidity equivalent to a 0.5 McFarland standard), and diluting this suspension 1:100 in Mueller-Hinton II (MH) broth (BD Biosciences, San Jose, CA, USA), achieving a final concentration of approximately 10^6^ CFU/ml. eBDs were serially 2-fold diluted in MH broth at concentrations ranging from 200 μg/ml to 0.02 μg/ml and pipetted into 96-well microtiter plates (Greiner Bio, Kremsmünster, Austria). The inoculum was added to the eBD dilutions 1:1, and the bacteria were incubated in a humidified incubator (5% CO_2_) at 37°C for 24 h. MIC values were determined as the lowest concentration of eBD at which there was no visible bacterial growth.

### Cells.

**(i) RK13 cells.** Rabbit kidney (RK13) cells were purchased from the American Type Culture Collection (ATCC; Manassas, VA, USA) and maintained in minimal essential medium (MEM) (Thermo Fisher Scientific) with 10% fetal calf serum (FCS; Thermo Fisher Scientific) and antibiotics.

**(ii) MDCK cells.** Madin-Darby canine kidney (MDCK) epithelial cells (ATCC) were maintained in MEM containing 1 mg/ml lactalbumin (Sigma-Aldrich) and antibiotics.

**(iii) EREC.** Primary equine respiratory epithelial cells (EREC) were isolated and cultured as described previously (26, 55).

**(iv) Equine monocytes, equine T lymphocytes, and equine polymorphonuclear cells.** Equine peripheral blood mononuclear cells (PBMC) and polymorphonuclear (PMN) cells were isolated as described previously (47, 56, 57).

### Viruses, antiviral assays, and virus binding assays.

**(i) Viruses.** Equine herpesvirus type 1 (EHV1), equine arteritis virus (EAV), and equine influenza virus (EIV) comprise the three most abundant viruses causing respiratory symptoms in the horse. A Belgian EHV1 isolate (isolate 03P37) was used in this study and originated from the blood taken from a paralytic horse during an outbreak in 2003 (58). In addition, the virulent RacL11 EHV1 strain, isolated from an aborted foal, was used (59). Construction of EHV1 RacL11 lacking gM or gp2 (RacL11 ΔgM and RacL11 Δgp2, respectively) has already been reported (36, 60). The absence of gM protein expression in RacL11 ΔgM-infected RK13 cells was verified using immunofluorescence staining (data not shown). The Belgian EAV 08P178 strain was isolated from a neonatal foal suffering from respiratory distress (61). The EHV1 and EAV strains were grown on RK13 cells and used at the 7th passage. Final passages were propagated in FCS-deprived medium to obtain a serum-free virus stock. Stock solutions were titrated onto RK13 cells. The A/equine/Kentucky/98 influenza A virus strain (H3N8) was propagated on eggs and titrated onto MDCK cells.

**(ii) Purification and DiO labeling of EHV1 for binding assays.** Virus purification and subsequent DiO labeling were performed as described previously (26, 62).

**(iii) Removal of N-linked glycans from EHV1 virion surfaces.** PNGase F (New England Biolabs, Ipswich, UK) removes complex, hybrid, and oligomannose N-glycosylations and was added to purified EHV1 virions for 12 h at a concentration of 25,000 U/ml, diluted in phosphate buffer, supplemented with 10% glycobuffer (New England Biolabs, Ipswich, UK). As a negative control, EHV1 virions were treated with 10% glycobuffer only, dissolved in phosphate buffer for 12 h.

**(iv) Antiviral assay on RK13 and MDCK cells.** Cells were grown to confluence in 24-well plates before thoroughly washing them with MEM to remove excess FCS. In a synchronized assay, the impact of eBD1, -2, and -3 on (i) direct virus inhibition, (ii) cell protection from subsequent viral infection, (iii) viral entry, and (iv) viral postentry was evaluated in different sets of wells. RK13 cells were used for EHV1 and EAV plaque assays, and MDCK cells were used for EIV plaque assays. (i) Direct virion inactivation was investigated by preincubating the 100× inoculum diluted in 10 mM phosphate buffer (PB) with eBD1, -2, and -3 at concentrations ranging from 0 to 100 μg/ml for 1 h at 37°C. Heparin treatment (100 U/ml; Leo) was included as a positive control for EHV1 binding inhibition. The inoculum was then diluted 1:100 in MEM in order to dilute the remnant eBD in the inoculum. The cells were inoculated with pretreated inoculum at a final multiplicity of infection (MOI) of 0.001 for 2 h at 4°C. (ii) The cells were pretreated with eBD1, -2, and -3 (0 to 100 μg/ml) diluted in MEM for 1 h at 37°C, prior to inoculation, to examine whether eBDs protect cells from subsequent viral infection. Before inoculation (MOI, 0.001; 2 h; 4°C), excess eBD1, -2, and -3 was washed away with MEM. Following virion binding at 4°C, nonadherent virus particles were removed by washing all cells 3 times on ice. (iii) eBD1, -2, and -3 (0 to 100 μg/ml) diluted in MEM were added to the cells during virus entry at 37°C (1 h). Following the viral entry step at 37°C (1 h), all cells were briefly exposed to 40 mM citrate buffer (pH 3) to destroy any virus particles that had not entered the cells. Finally, all cells were covered with a solution containing 50% 2× MEM (Thermo Fisher Scientific) and 50% 1.88% carboxymethylcellulose (Sigma-Aldrich). In a final set of wells, the latter solution was supplemented with eBD1, -2, and -3 at concentrations ranging from 0 to 100 μg/ml to evaluate their effect during (iv) postentry. Ganciclovir (10 μg/ml; Cymevene; Roche), a herpesvirus kinase-activated nucleoside analogue terminating polymerization of the viral DNA chain, was included as a positive control for the inhibition of EHV1 replication. EHV1 and EAV plaques were visualized at 48 h after inoculation by fixing and staining the RK13 cell monolayers with a mixture of 0.5% crystal violet (Sigma-Aldrich), 3% formaldehyde, and 20% ethanol. MDCK cell monolayers were fixed in 4% paraformaldehyde at 4°C for 15 min. Immunoperoxidase staining of influenza A virus proteins was performed using the mouse monoclonal HB-65 antibody, diluted in Tris-buffered saline with 0.1% saponin and 2% NGS, followed by incubation with a 5% aminoethyl carbazole (AEC) solution, supplemented with 0.025% H_2_O_2_. The total number of EHV1 and EAV plaques per well and the total number of EIV-positive MDCK cells per well were counted using an inverted light microscope. Images were taken with a CTL ImmunoSpot F-419 camera (Cellular Technology Limited, Cleveland, OH, USA), and the diameter of the EHV1 plaques was measured using ImageJ software (U.S. National Institutes of Health, Bethesda, MD, USA). The viability of the cells was assessed by ethidium monoazide bromide (EMA) staining, ensuring that the treatment with eBDs did not cause a significant cell loss.

**(v) Antiviral assay on EREC.** Cells were grown to full differentiation in a transwell cell culture system prior to inoculation with EHV1 (at basolateral surfaces, MOI, 0.2) or EIV (at apical surfaces, MOI, 2). Similarly, as described above for RK13 and MDCK cells, the impact of eBD1, -2, and -3 on (i) direct virion inhibition, (ii) cell protection from subsequent viral infection, (iii) viral entry, and (iv) viral postentry was evaluated. At 18 h postinoculation, the cells were fixed in methanol for 20 min at −20°C and stained for EIV or EHV1 proteins by immunofluorescence. Using confocal microscopy, the total number of EHV1 plaques in 5 random fields of approximately 3 × 10^4^ cells per insert was counted. Plaque diameter was measured for 10 individual plaques using the Leica confocal software package. The total number of EIV-positive cells in 5 random fields of approximately 1.5 × 10^4^ cells per insert was counted. The viability of the cells was assessed by EMA staining, ensuring that the treatment with eBD1, -2, and -3 did not cause a significant cell loss.

**(vi) EHV1 binding assay.** To characterize the attachment of EHV1 virions to RK13 cells and EREC upon treatment with different eBDs, direct binding studies were carried out with DiO-labeled EHV1 particles. Following virus or basolateral EREC pretreatment with 0 or 100 μg/ml eBD1, -2, and -3, diluted in PB, cells were chilled on ice for 5 min and washed 3 times with cold PBS. Pretreated DiO-labeled virus particles were diluted 1:100 before inoculating RK13 cells at an MOI of 0.01 for 2 h at 4°C. Pretreated EREC were inoculated at the inverted basolateral surfaces with DiO-labeled EHV1 particles at an MOI of 0.2 for 2 h at 4°C. Nonadsorbed virus was removed by washing the cells 3 times with cold PBS. The cells were then fixed for 10 min in 1% paraformaldehyde. Nuclei were counterstained with Hoechst 33342 for 10 min at room temperature, and slides were mounted with glycerol-DABCO. The percentage of cells with bound EHV1 particles was calculated based on the number of cells with viral particles bound on the plasma membrane of 300 randomly selected cells. The number of virus particles attached per cell was calculated based on the number of particles attached at the plasma membrane of 10 random EHV1-positive cells. For each cell, the entire plasma membrane was screened for the presence of virus particles by the use of confocal microscopy.

### Induction of β-defensin expression in EREC in response to EHV1 and EIV infection.

At 18 h postinoculation (EHV1 MOI, 0.2 at basolateral surfaces; EIV MOI, 2 at apical surfaces), EREC were fixed in 1% paraformaldehyde for 10 min and simultaneously stained for EIV or EHV1 proteins and eBD1, -2, and -3 by immunofluorescence. In EHV1- and EIV-inoculated cells, infected cells were distinguished from noninfected cells based on immunofluorescence staining of viral proteins. The mean percentage of the eBD fluorescent signal was calculated on 5 complete Z-stack confocal images using ImageJ software (U.S. National Institutes of Health, Bethesda, MD, USA). Finally, the mean percentage of the fluorescent signal (i.e., the eBD signal) from the mock-inoculated cells was compared to that of EHV1- or EIV-inoculated cells, with the last two groups containing infected as well as noninfected areas.

### Chemotaxis assay.

The chemotactic activity of eBD1, -2, and -3 for equine polymorphonuclear cells, CD172a^+^ monocytic cells, and CD3^+^ T lymphocytes was determined using a 96-well Boyden chamber containing 5-μm-pore-size membranes (CytoSelect; Cell Biolabs, San Diego, CA, USA), following the manufacturer’s instructions. Cell migration values were reported as relative fluorescence units (RFU), measured at 480/520 nm, using a Fluoroskan Ascent FL (Thermo Fisher Scientific) plate reader. Finally, the background RFU from RPMI supplemented with eBD1, -2, and -3, 10 mM PB (negative control), or 10% FCS (positive control) were subtracted from the respective cell migration values.

### Statistical analyses.

Significant differences (*P* < 0.05) between different treatments with or without eBDs and between mock-, EHV1-, and EIV-inoculated cells were identified by analysis of variance (ANOVA), followed by a two-sided Dunnett’s *post hoc* test. If homoscedasticity of the variables was not met, as assessed by Levene’s test, the data were log transformed prior to ANOVA. The normality of the residuals was verified by using the Shapiro-Wilk test. If the variables remained heteroscedastic or normality was not met after log transformation, a Kruskal-Wallis test, followed by Mann-Whitney’s *post hoc* test, was performed. All analyses were conducted in IBM SPSS Statistics for Windows, version 25.0 (IBM Corp., Armonk, NY, USA).

### Data availability.

The raw data supporting the conclusions presented in this article will be made available by the authors, without undue reservation, to any qualified researcher.
